# Coffee Berry Borer Joins Bark Beetles in Coffee Klatch

**DOI:** 10.1371/journal.pone.0074277

**Published:** 2013-09-20

**Authors:** Juliana Jaramillo, Baldwyn Torto, Dickson Mwenda, Armin Troeger, Christian Borgemeister, Hans-Michael Poehling, Wittko Francke

**Affiliations:** 1 Institute of Plant Diseases and Plant Protection, Leibniz University Hannover, Hannover, Germany; 2 International Center of Insect Physiology and Ecology (*icipe*), Nairobi, Kenya; 3 Institute of Organic Chemistry, University of Hamburg, Hamburg, Germany; AgroParisTech, France

## Abstract

Unanswered key questions in bark beetle-plant interactions concern host finding in species attacking angiosperms in tropical zones and whether management strategies based on chemical signaling used for their conifer-attacking temperate relatives may also be applied in the tropics. We hypothesized that there should be a common link in chemical signaling mediating host location by these Scolytids. Using laboratory behavioral assays and chemical analysis we demonstrate that the yellow-orange exocarp stage of coffee berries, which attracts the coffee berry borer, releases relatively high amounts of volatiles including conophthorin, chalcogran, frontalin and sulcatone that are typically associated with Scolytinae chemical ecology. The green stage of the berry produces a much less complex bouquet containing small amounts of conophthorin but no other compounds known as bark beetle semiochemicals. In behavioral assays, the coffee berry borer was attracted to the spiroacetals conophthorin and chalcogran, but avoided the monoterpenes verbenone and α-pinene, demonstrating that, as in their conifer-attacking relatives in temperate zones, the use of host and non-host volatiles is also critical in host finding by tropical species. We speculate that microorganisms formed a common basis for the establishment of crucial chemical signals comprising inter- and intraspecific communication systems in both temperate- and tropical-occurring bark beetles attacking gymnosperms and angiosperms.

## Introduction

Phytophagous insects recognize specific olfactory signals in order to find their hosts against the ‘background noise’ caused by environmental odours This specificity in olfactory signaling is typically defined by its quantity and/or quality, which in turn is governed by relative proportions of components in the signal [1]. As a result of these interactions, some plants have evolved to mimic the odor bouquet of certain insects to cause avoidance behaviour in herbivores [2], as demonstrated for bark beetles whose sensory detection system allows them not only to detect host volatiles, but also to avoid non-host volatiles [3]–[6].

Scolytinae beetles (Coleoptera: Curculionidae: Scolytinae) are important pests of forest trees and crops, and their interactions with host plants represent excellent models for behavior studies and chemical signaling because of their basic biology and wide distribution. Within Scolytinae, bark beetles mainly feed on bark and phloem, occasionally on seeds, acquiring most of their nutrients from dead plant tissue [7], [8]. Ambrosia beetles (Scolytinae and Platypodinae), on the other hand, attack xylem tissue of their hosts and are typically associated with symbiotic microorganisms, particularly with fungi but also with bacteria. Ambrosia beetles are found largely in wet tropical forests [9].

For the majority of bark beetles whose chemical signaling systems have been studied, host location has been demonstrated in species that attack coniferous trees in temperate parts of the world [10], [11]. Odors attractive to these insects emanate predominantly from a number of gymnosperm hosts [8] and from a few species of angiosperms [12]. Because in the natural habitat these host tree species are usually highly scattered throughout mixed species forests and distributed unevenly in space and time [13], host colonization in bark beetles like *Dendroctonus*, *Ips*, *Scolytus*, *Pityogenes* and *Trypodendron* proceeds via multiple mechanisms including recognition of intra- and interspecific semiochemicals, host kairomones and avoidance of non-host volatiles [5], [14]–[18]. Both host monoterpenes (e.g. α-pinene and myrcene) and fatty acids may be utilized as precursors for the biosynthesis of pheromones, for host location, and as indicators for the suitability of a host in this group of bark beetles.

For the group that has a symbiotic relationship with microorganisms, for example, *Xylosandrus* and *Xyleborus* spp., host colonization seems to be solely dependent on host odors, comprising mainly terpenes and fermenting odors such as ethanol for attraction [19]–[22].

The coffee berry borer *Hypothenemus hampei* (Coleoptera: Scolytinae) is a tropical pest, with its primary hosts being *Coffea arabica* and *C. canephora*. Chemical signaling between *H. hampei* and its host is not well understood despite its economic importance – annual losses surpassing US $ 500 million and 25 million farmers affected worldwide. Several studies have attempted to directly or indirectly examine the composition of volatiles produced by the coffee berries, aiming at their application in integrated pest management. Currently, a 1∶1 mixture of methanol and ethanol is widely used in trapping devices [23]–[26]. Yet, even though *H. hampei* capture rates can be quite high, they still represent a low percentage of the total pest population in a plantation and thus fail to serve as a mass-trapping device.

It was found that in the coffee berry borer-host plant system, unlike previously reported for bark beetles attacking coniferous trees, volatiles contributing to the attractive signal were mainly non-terpenoid compounds [27]–[29], however, the ecological and evolutionary links between *H. hampei* and other Scolytids in terms of the semiochemicals mediating host location is still unknown. Furthermore, a key and unanswered question in bark beetle-plant interactions is how species occurring in the tropical zones that attack angiosperms find their hosts and whether management strategies based on chemical signalling used for their conifer-attacking temperate relatives may be applied to their tropical counterparts. These and other important gaps in our knowledge of bark beetle chemical ecology need to be addressed in order to fully understand the evolution of chemical signaling in these beetles.

We hypothesized a common link in chemical signaling mediating host location in scolytids. The rationale behind this hypothesis is that in evolutionary terms this link should be driven by the biology and ecology of the beetle and their hosts they interact with.

Here, we present evidence using gas chromatography combined with electroantennography and mass spectrometric analyses as well as laboratory olfactometer and wind tunnel assays that coffee berries produce volatile compounds that are known pheromones associated with coniferous Scolytinae and which serve as kairomones for host location by the tropical relative, the coffee berry borer.

## Results

### Headspace Volatiles and Identification of Electrophysiologically Active Compounds

**Figure 1:**
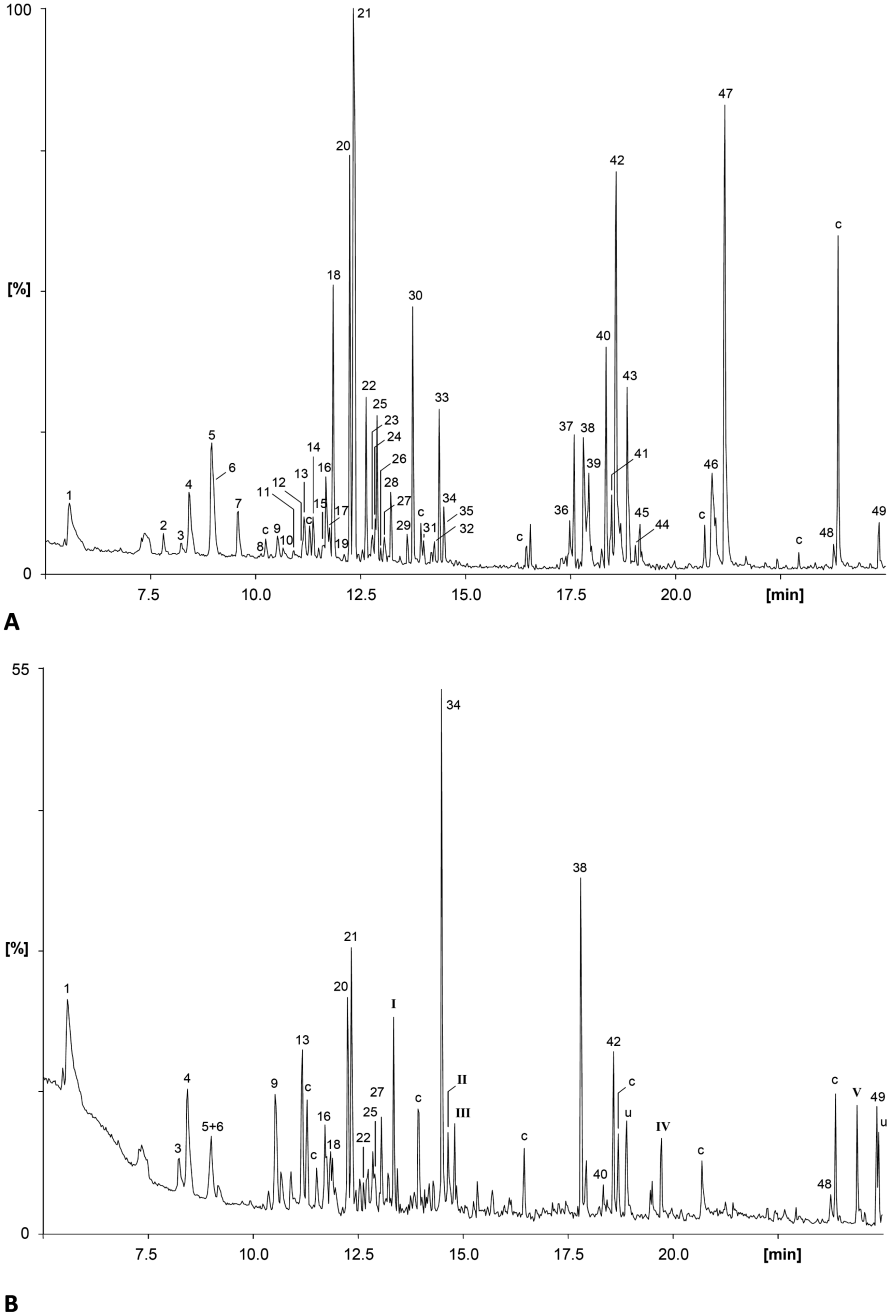
TIC-chromatogram of ripe *Coffea arabica* berries (A) and TIC-chromatogram of green *Coffea arabica* berries (B). The following VOCs were not found in the yellow coffee berries: I: (*E*)-4,8-dimethylnona-1,3,7-triene, II: methyl salicylate, III: 1-methylethyl benzoate, IV: E-dendrolasin, V: 1-methylethyl hexadecanoate. Peak numbers are identical to those in Table 1; c = contaminant, present also in blank runs, u = unknown.

About 50 components, mainly oxygen-containing compounds, were identified among the volatile organic compounds (VOCs) emitted by the yellow-orange exocarp stage of coffee berries (Fig. 1A and Table 1). Comparison of the pattern of VOCs emitted by yellow-orange exocarp and green coffee berries (Fig. 1B) reveals that the latter produce relatively small amounts of a much less complex bouquet, which, in a way, supports Matthieu et al. [28] who report the identification of only one compound in green berries and ten in red ones.

**Table 1:** Volatile compounds from the headspace of yellow-orange exocarp coffee berries (*C. arabica*).

#	RT [min]	RI	chemical name	rel. Int. ripe berries	rel. Int. green berries
1	5.57	763	Toluene	19	68
2	7.81	849	4-Hydroxy-4-methylpentane-2-one	3	
3	8.22	865	Ethylbenzene	2	14
4	8.42	872	C_2_-Benzene	14	50
5	8.96	893	Styrene	29	16
6	9.02	896	C_2_-Benzene	tr	14
7	9.58	921	Anisol	8	
8	10.12	947	Frontalin	tr	
9	10.52	966	C_3_-Benzene	4	44
10	10.72	976	2-Methoxy-3-methylpyrazine^a^	tr	
11	10.90	984	C_3_-Benzene	1	
12	11.11	994	6-Methyl-5-heptene-2-one^a^	tr	
13	11.15	996	C_3_-Benzene	7	43
14	11.37	1008	α-Phellandrene	5	
15	11.62	1022	3-Ethyl-4-methylpentanola,b	tr	
16	11.67	1024	*m*-Methylanisol	5	19
17	11.76	1029	*p*-Cymene	1	12
18	11.84	1034	β-Phellandrene	27	13
19	12.11	1050	1,6-Dioxaspiro[4.5]decane^b^	1	
20	12.25	1057	Methyl 3-ethyl-4-methylpentanoate^b^	34	51
21	12.34	1062	(5*R*,7*S*)-Conophthorin	100	65
22	12.63	1079	*trans*-Linalool oxide (furanoid)	13	5
23	12.79	1088	Chalcogran^a^	2	
24	12.85	1091	Chalcograna,b	tr	
25	12.90	1094	*cis*-Linalool oxide (furanoid)	9	8
26	12.99	1099	3-(1-Methylethyl)-2-methoxypyrazine	1	
27	13.08	1105	Linalool	3	3
28	13.21	1113	6-Ethenyldihydro-2,2,6-trimethyl-2*H*-pyran-3(4*H*)-one	7	
**I**	13.35	1121	(*E*)-4,8-Dimethylnona-1,3,7-triene		35
29	13.61	1138	(5*S*,7*S*)-Conophthorin^b^	3	
30	13.75	1147	1-Terpineol	21	
31	13.99	1162	Isomenthone	1	
32	14.26	1179	3-*sec*-Butyl-2-methoxypyrazine	2	
33	14.38	1186	3-Isobutyl-(2-Methylpropyl-2-methoxypyrazine	12	
34	14.49	1193	Naphthalene	6	100
35	14.51	1195	Dillether	5	
**II**	14.64	1203	Methyl salicylate		15
**III**	14.80	1214	1-Methylethyl benzoate		15
36	17.49	1407	Sativene^c^	6	
37	17.58	1414	Unknown sesquiterpene	10	
38	17.80	1432	α-Cedrene	19	69
39	17.94	1442	Acora-3,5-diene^c^	11	
40	18.34	1474	Prezizaene^c^	21	6
41	18.47	1485	β-Acoradiene^c^	7	
42	18.59	1493	α-Neocallitropsene^c^	45	34
43	18.86	1515	α-Muurolene^c^	19	
44	19.03	1530	Unknown sesquiterpene	2	
45	19.15	1539	δ-Cadinene^c^	3	
**IV**	19.73	1587	(*E*)-Dendrolasin		21
46	20.87	1687	Oxygenated sesquiterpene	19	
47	21.18	1715	Oxygenated sesquiterpene	59	
48	23.76	1964	Hexadecanoic acid	3	7
**V**	24.39	2029	1-Methylethyl hexadecanoate		22
49	24.86	2079	Kaurene	4	24

a rel. Integ below 0.05.

b synthesized in Hamburg.

c sesquiterpene, tentative structure.

tr trace.

c contaminant.

The major component among the yellow-orange exocarp coffee berry VOCs was found to be the spiroacetal conophthorin which had been identified earlier as a component of the aroma of green Mexican coffee [30]. By enantioselective gas chromatography using a modified cyclodextrin as the stationary phase, the two enantiomers of conophthorin were very well resolved showing an α-value of tr(5*S*,7*S*):tr(5*R*,7*R*) = 1.13. Coinjection proved the natural product to show (5*S*,7*S*)-configuration. The compound was accompanied by very small amounts of its (5*R*,7*S*)-stereoisomer. A second major compound iamongn the coffee berry VOCs was methyl 3-ethyl-4-methylpentanoate. In addition, identified coffee berry VOCs associated with Scolytinae chemical ecology were chalcogran and frontalin (Table 1). An unsubstituted spiroacetal, 1,6-dioxaspiro[4.5]decane structurally very close to conophthorin, represents a new natural product. Other important minor constituents were the pyrazines 2-methoxy-3-methylpyrazine, 3-isopropyl-2-methoxypyrazine, 3-*sec*.-butyl-2-methoxypyrazine, and 3-isobutyl-2-methoxypyrazine, (Table 1 and Fig. 1A). Some of the pyrazines account for the typical smell of coffee berries [30], in addition to the VOCs found in extracts of charcoal filters used for head space adsorption (see Table 1). Application of solid phase microextraction [31] SPME led to the identification of acetoin and stereoisomers of 2,3-butandiols. Differences in analytical results of plant VOCs as observed in the present study as compared to earlier work [27]–[30] may be due to dissimilar techniques used to collect and analyze VOCs, and potential differences in the plant material used (variety/clone, physiological state, growth phase etc).

**Figure 2:**
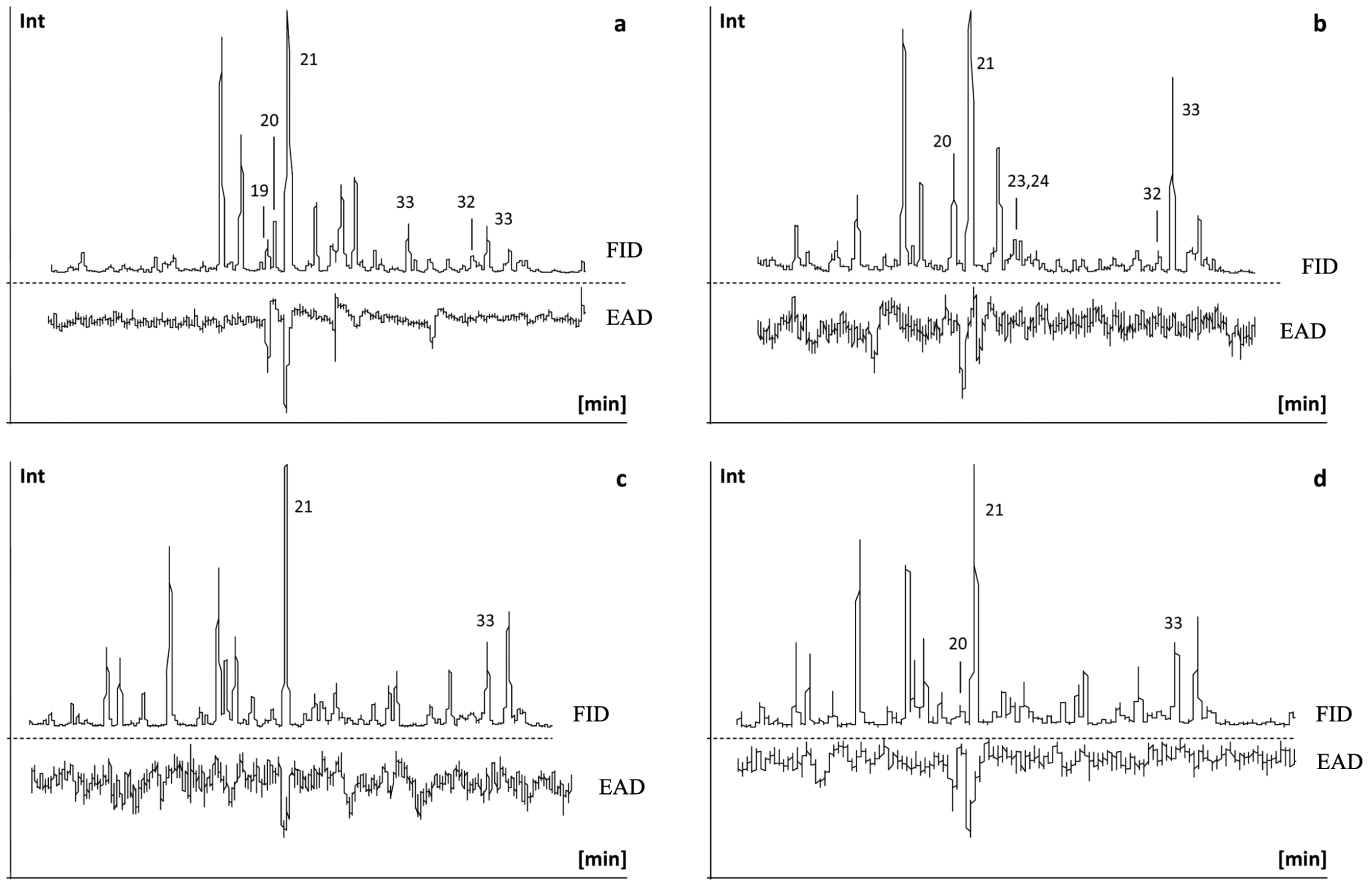
Four GC/EAD runs of extracts of yellow coffee berries (*C. arabica*) using antennae of females of *Hypothenemus hampei*. Peak numbers are the same as in Figures 1A,B and in Table 1. EAD = Electroantennographic detector, FID = Flame ionization detector.

Coupled GC/EAD analysis revealed that among the coffee berry volatiles only (*5S,7S*)-conophthorin and 1,6-dioxaspiro[4.5]decane consistently elicited EAG activity using antennae of *H. hampei* females (Fig. 2). In order to exclude that the negative/not conclusive EAG results obtained with some of the compounds were due to their low concentration in the coffee berry extract, further dose response GC/EAD-runs were carried out using authentic reference samples of *rac*.-chalcogran, frontalin, α-pinene, methyl 3-ethyl-4-methylpentanoate, 3-*sec*.butyl-2-methoxypyrazine, and 3-isobutyl-2-methoxypyrazine, at different concentrations (10 to 100 ng/µl). Positive EAG responses were recorded for frontalin and α-pinene at a concentration of 50 ng/µl. No antennal activity to *rac*.-chalcogran, methyl 3-ethyl-4-methylpentanoate, or any of the identified pyrazines was found.

Surprisingly, no EAG responses were detected for the compounds and concentrations previously reported by Mendesil et al. [27] (i.e., methylcyclohexane, nonane, ethylbenzene, (*R*)-limonene, 1-octen-3-ol, and (R)-3-ethyl-4-methylpentanol).

### *H. hampei* Responses to Host Volatiles:Y-tube Olfactometer Assays

Dose response experiments were carried out using a y-tube olfactometer to elucidate the behavioural mediating capacity of candidate compounds. To test for directional bias of the insects, the responses of *H. hampei* females were tested with no odor sources placed in the olfactometer arms. The majority of *H. hampei* females (N = 40) did not respond after 15 minutes and failed to choose either arm of the olfactometer. The remaining females (N = 20) showed no significant preference for any of the arms (χ^2^_1_ = 0.1 P = 0.7518).

**Figure 3:**
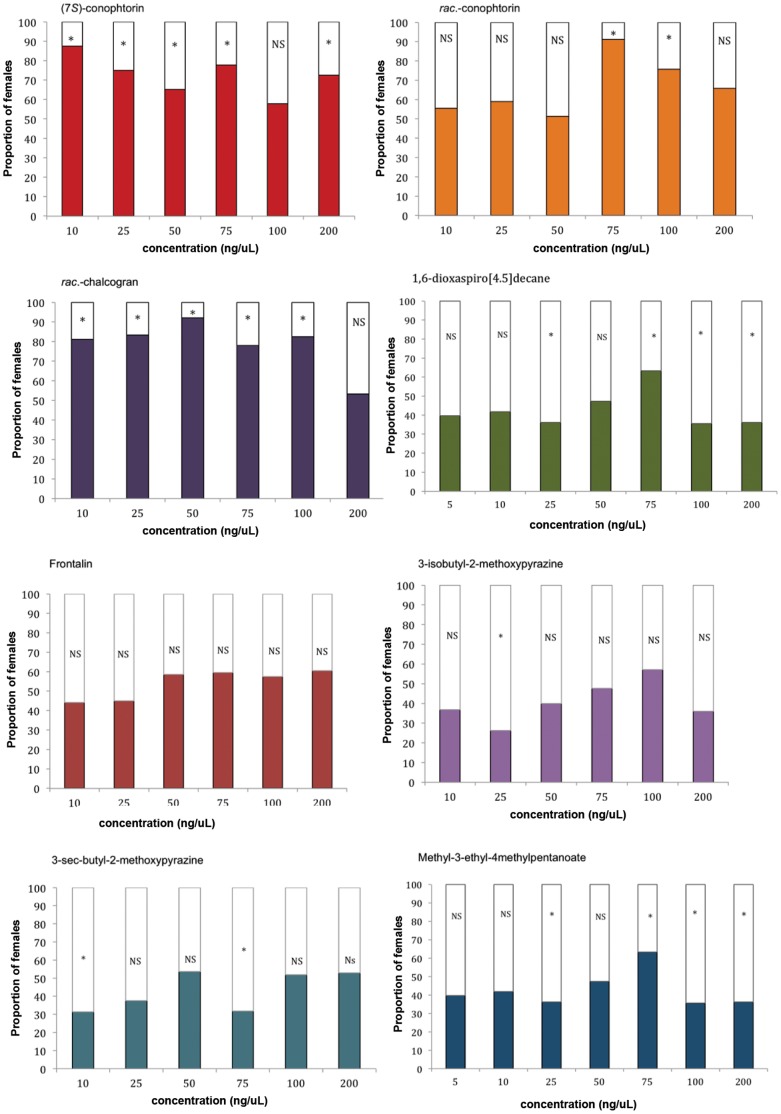
Responses of walking *Hypothenemus hampei* females (60 individuals per concentration/test) in a Y-tube olfactometer to different compounds and concentrations (in colours). Bars denoted by asterisks indicate a significant preference for that treatment (P<0.05).

*H. hampei* females exhibited a significant preference for (5*S*,7*S*)-conophthorin (10 ng/µl; 87.5%; N = 47), *rac*.-conophthorin (75 ng/µl; 91.2%; N = 56) and *rac*.-chalcogran (50 ng/µl; 92.1%; N = 53) compared to the control across all concentrations tested (Fig. 3; Table S1). On the other hand, no significantly positive behavioral responses were recorded for 1,6-dioxaspiro[4.5]decane and *rac*.-frontalin or 3-*sec*.-butyl-2-methoxypyrazine, and 3-isobutyl-2-methoxypyrazine (Fig. 3; Table S1). For methyl 3-ethyl-4-methylpentanoate the behavioral responses were not conclusive, with significant attraction at 75 ng (N = 60) and somehow avoidance at concentrations 25 (N = 58), 100 (N = 59) and 200 (N = 58) (Fig. 3). In all cases except for (5*S*,7*S*)-conopthorin and *rac*.-frontalin, there seems to be an increase in response with the dose and then a decrease at concentrations higher than 100 ng/µl, which suggests a possible inhibition at high concentrations.

**Figure 4:**
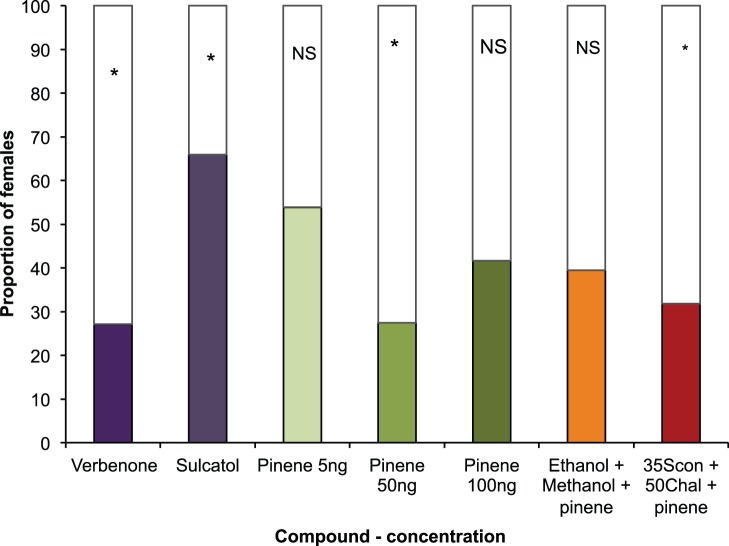
Responses of individual walking *Hypothenemus hampei* females (60 individuals per concentration/test) in a Y-tube olfactometer to different compounds (in black) against control (white). Bars denoted by asterisks indicate a significant preference for that treatment (P<0.05).

Verbenone and α-pinene (50 ng/µl) were significantly unattractive to *H. hampei* females (χ^2^_1_ = 10.0835 P = 0.0015; N = 48; χ^2^_1_ = 10.3725 P = 0.0013; N = 51, respectively). Neither attraction nor avoidance was recorded for α-pinene at 5 ng/µl (χ^2^_1_ = 0.3077 P = 0.5791; N = 52) or α-pinene at 100 ng/µl (χ^2^_1_ = 1.00 P = 0.3173; N = 46). Interestingly, α-pinene seemed to have an antagonistic effect on the attractive mixture ethanol (300 ng/µl)+methanol (700 ng/µl) (χ^2^_1_ = 1.6842 P = 0.1944; N = 38), as well as on the mixture of (5*S*,7*S*)-conophthorin (35 ng/µl)+*rac*.-chalcogran (50 ng/µl) (χ^2^_1_ = 5.8182 P = 0.0159; N = 44, respectively) (Fig. 4). Sulcatol had a marginally significant attractive effect on *H. hampei* females (χ^2^_1_ = 4.4545 P = 0.0348; N = 44) (Fig. 4).

### *H. hampei* Responses to Host Volatiles: Wind Tunnel Assays

**Figure 5:**
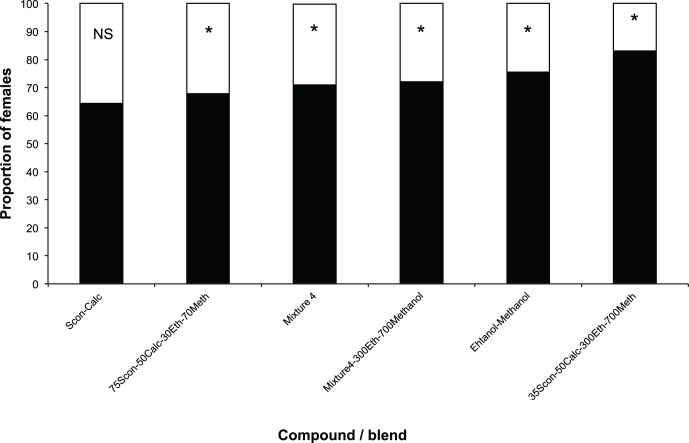
Responses of individual *Hypothenemus hampei* females (225 individuals per compound/mixture) in windtunnel trials to different mixtures (in black). Bars denoted by asterisks indicate a significant preference for that treatment (P<0.05).

**Figure 6:**
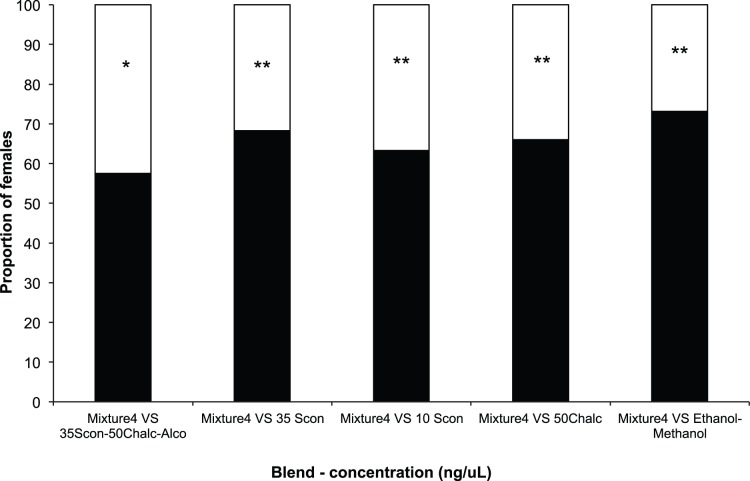
Responses of individual *Hypothenemus hampei* females (225 individuals per compound/mixture) in windtunnel trials to the most attractive compounds against the most attractive compounds to *H. hampei* females. Bars denoted by asterisks indicate a significant preference for that treatment (P<0.05).

**Figure 7:**
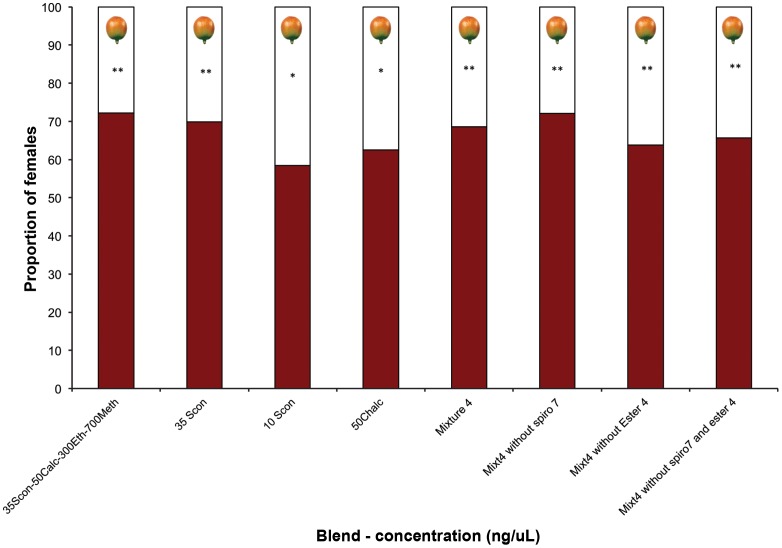
Responses of individual *Hypothenemus hampei* females (225 individuals per compound/mixture) in windtunnel trials to the most attractive compounds (in red) versus non-infested fresh yellow coffee berries (in white). Spiro 7: 1,6-dioxaspiro[4.5]decane; Ester 4: methyl 3-ethyl-4-methylpentanoate. Bars denoted by asterisks indicate a significant preference for that treatment (P<0.05).

The number of females making a positive choice in the wind tunnel was significantly higher for all compounds/blends tested, with the highest percentage of *H. hampei* females reacting positively to the blend of (5*S*,7*S*)-conophthorin+*rac*.-chalcogran+ethanol+methanol (N = 221, and N = 218, respectively for lower and higher concentrations of alcohols) (Fig. 5; Table S2). Subsequently, when the most attractive compounds/blends were tested against each other, in all tests mixture 4 (see Table 2) was more attractive than the pure compounds: *rac*.-chalcogran (50 ng/µl) (χ^2^_1_ = 21.15 P = <0.0001; N = 206), (5*S*,7*S*)-conophthorin (10 and 35 ng/µl) (χ^2^_1_ = 14.635 P = 0.0001: N = 207; χ^2^_1_ = 28.099 P = <0.0001; N = 214), and the mixture of ethanol (300 ng/µl)+methanol (700 ng/µl) (χ^2^_1_ = 43.0299 P = <0.0001; N = 201) (Fig. 6). Although significantly different (χ^2^_1_ = 4.6425 P = 0.0312; N = 207), mixture 4 was only slightly more attractive (57.5%) than the mixture of (5*S*,7*S*)-conophthorin (35 ng/µl)+*rac*.-chalcogran (50 ng/µl)+ethanol (300 ng/µl)+methanol (700 ng/µl) (42.5%) (Fig. 6). In these tests the most attractive compounds/blends tested were always significantly more attractive than fresh coffee berries (Fig. 7; Table S3).

**Table 2:** Chemicals, blends and concentrations used for behavioral olfactometer assays with *Hypothenemus hampei* females.

Compound	Concentrations tested (ng/µl)	EAD response
(5*S*,7*S*)-Conophthorin	10, 25, 50, 75, 100, 200	Positive
*rac*.-Conophthorin	10, 25, 50, 75, 100, 200	Not conclusive
*rac*.-Frontalin	10, 25, 50, 75, 100, 200	Positive
1,6-Dioxaspiro[4.5] decane*	5, 10, 25, 50, 75, 100, 200	Positive
*rac*.-Chalcogran	10, 25, 50, 75, 100, 200	Not conclusive
Methyl 3-ethyl-4-methylpentanoate*	5, 10, 25, 50, 75, 100, 200	Not conclusive
3-*sec*-Butyl-2-methoxypyrazine	10, 25, 50, 75, 100, 200	Not conclusive
3-Isobutyl-2-methoxypyrazine	10, 25, 50, 75, 100, 200	Not conclusive
Mixture 4: (5*S*,7*S*)-conophthorin +1,6-dioxaspiro[4.5]decane+methyl 3-ethyl-4-methylpentanoate+*rac*.-chalcogran	35+1+1.5+1.2	Not tested
Verbenone (ConTech Inc. USA)	Commercial formulation	Not tested
Sulcatol (ConTech Inc. USA)	Commercial formulation	Not tested
α-Pinene	5, 50, 100	Positive at 50 ng
α-Pinene+ethanol+methanol	50+300+700	Not tested
α-Pinene+(5*S*,7*S*)-conophthorin+*rac*.-chalcogran	50+35+50	Not tested

* No previous available information on the potential behavioral activity of these compounds on *H. hampei*. EAD responses: Positive refers to a repeatable positive response to the compound; not conclusive: refers to some antenna giving positive responses but not consistent repeatable responses were recorded with all the antennae tested for a specific compound/dose.

## Discussion

### Overlap of Odor Bouquets: Coffee Berries and Scolytinae

This study represents the first in-depth identification of volatile organic compounds (VOCs) from coffee berries that elicit responses from the coffee berry borer *H. hampei.* We found that coffee berries (*C. arabica*) produce compounds identical with pheromones of coniferous Scolytinae which attack coniferous trees and which serve as kairomones for host location by the tropical relative, the coffee berry borer.

The spiroacetal conophthorin and methyl 3-ethyl-4-methylpentanoate were identified as the dominant components among the coffee berry VOCs, in contrast to previous studies on the chemical composition of coffee berries and chemical ecology of the coffee berry borer [27]–[29].

Several of the compounds identified in this study such as frontalin, *rac*.-chalcogran, sulcatone and conophthorin are associated with Scolytinae chemical ecology. For example, the bicyclic acetal frontalin, whose presence in coffee is being reported for the first time in the present study, is a well-known component of communication systems of Scolytinae [14]; as a typical pheromone of bark beetles, especially of *Dendroctonus* spp. [32], as a spacing factor [33], and having a function of aggregation or anti-aggregation pheromone in various Scolytinae species [14]. In vertebrates, it is a component of the sex pheromone of the male Asian elephant *Elephas maximus* [34] and the female African elephant *Loxodonta africana* [35].

The spiroacetal chalcogran is an aggregation pheromone of bark beetles of the genus *Pityogenes* [36] and has been reported as part of the VOCs emitted by angiosperm trees [3], [37].

The terpenoid 6-methyl-5-hepten-2-one (sulcatone), frequently found as a VOC of plants is structurally close to the corresponding alcohol, sulcatol, an aggregation pheromone known from bark beetles of the genus *Gnathotrichus* [38]. Conophthorin is also relatively widespread and serves as a behavior mediating volatile produced by several bark beetle species [36]. In some species it acts as an aggregation pheromone and sex attractant, whereas in others it is a strong repellent. Conophthorin has also been described as a component of flower scent and as a constituent of tree VOCs [39] and coffee berries [30]. In addition to conophthorin we identified the structurally related unsubstituted spiroacetal 1,6-dioxaspiro[4.5]decane, which is a new natural product. The plotted 70 eV EI-mass spectrum of the compound is given as supporting information (Figure S1). Also identified were methyl 3-ethyl-4-methylpentanoate, previously identified from *Formica* ants [40], and trace amounts of its reduction product, the corresponding alcohol, 3-ethyl-4-methylpentanol. This alcohol is part of the sex attractant queen pheromone of ants of the genus *Polyergus* [41], [42] and a component of an attractive blend of VOCs previously identified for the coffee berry borer [27]. In coupled GC/EAD analysis of VOCs obtained by head space collection of coffee berries conophthorin and its unsubstituted derivative elicited strong EAG responses in antennae of *H. hampei.* At the naturally occurring very low concentrations of the other scolytid-associated volatiles no electrophysiological response was found, however, at higher concentrations, antennae of the coffee berry borer detected all these VOCs.

### Responses of the Coffee Berry Borer to Host Volatiles

In our olfactometer and wind tunnel assays, females of *H. hampei* were attracted to scolytid-associated VOCs. In dose response assays, when tested individually we found that (5*S*,7*S*)-conopthorin, *rac*.-conophthorin and *rac.*-chalcogran strongly attracted *H. hampei* females, while 1,6-dioxaspiro[4.5]decane, *rac*.-frontalin, methyl 3-ethyl-4-methylpentanoate and sulcatol elicited moderate to weak attraction compared to the control. In contrast, *H. hampei* females avoided α-pinene and verbenone. These terpenes are long known as inhibitors for various species of Scolytinae [43], [44]. Our results are consistent with those of Burbano et al. [45], who also found verbenone and α-pinene to have a repellent effect on the bark beetle *Xylosandrus compactus* (Eichhoff) that attacks coffee in Hawaii. These results reflect differences in detection thresholds to individual components in the blend of VOCs, which would determine whether a host is attractive or unattractive, confirming previous findings on the function of semiochemicals in inter-specific interactions in bark beetles. Similarly, we found that the pattern of female response to different blends of these compounds was similar to that of the individual compounds. While some blends were attractive, others were unattractive or were avoided (Figures 3, 4, 5; Table S1–S2). The highest attraction was obtained with a combination of (5*S*,7*S*)-conophthorin and *rac*.-chalcogran. It appears that the presence of these two compounds in the host may significantly contribute to the attraction of *H. hampei*.

Our findings that the coffee berry borer detects these compounds just like other Scolytinae could be explained from an ecological standpoint. In ecological terms, there is overwhelming evidence indicating that conifer-attacking Scolytinae species not only detect and orient to their aggregation pheromones and host volatiles, but also are able to perceive and behaviorally avoid VOCs from non-host angiosperm trees, typically green leaf volatiles (GLV), some C8-alcohols, and the spiroacetal conophthorin [5], [17], [39], [46]–[51]. Conophthorin is probably the best studied non-host volatile found in the bark of several angiosperm species in Europe and in North America [15], [36], [39], [50]. It may, therefore, seem adaptive, evolutionary speaking, for Scolytinae species whose habitats are mixed species forests to be able to quickly and effectively discriminate between hosts and non host trees [52]. On the other hand, the habitat of *H. hampei*’s main hosts, *C. canephora* and *C. arabica*, is the understory of forests of tropical Africa [53]. Given our results, we hypothesize that, as previously found for conifer-attacking Scolytinae, the coffee berry borer also uses non-host VOCs (in this case monoterpenes and other components of gymnosperms) to find its way in mixed forests, whereas it uses conophthorin and chalcogran to locate its hosts, which follows the opposite mechanism used by its relatives attacking gymnosperms. Thus, although our results portray a general phenomenon in Scolytinae, it appears that different species have evolved to use different mechanisms to locate their hosts. Previous studies report that angiosperm-attacking Scolytinae such as *Xylosandrus crassiusculus*, which avoid monoterpenes from conifers [54], [55], are attracted to the non-host volatile conophthorin (Nicole van der Laan-Hannon; Hardwood Tree Improvement and Regeneration Center Purdue University, USA, pers. comm.).

Noteworthy is the response of *H. hampei* to some of the compounds combined with ethanol and methanol. Because the biology of this insect suggests a close association with its host and its lineage with microorganisms, it would certainly gain from being able to detect fermenting odors since microbial infection of the berries during feeding would lead to the release of corresponding scent. As such, it is not surprising that *H. hampei* is attracted to the synthetic blend of a few of these VOCs combined with ethanol and methanol. Methanol and ethanol are products associated with wood decay and are components of the bouquet used by some ambrosia beetles for host detection acting synergistically with other attractive compounds in several species e.g. [55]. Interestingly, addition of α-pinene at one concentration appeared to inhibit the attractive effect of the blend of ethanol and methanol and the blend comprising (5*S*,7*S*)-conophthorin and *rac*.-chalcogran. The behavioral effect shown by the addition of α-pinene further supports our hypothesis of the use of host and non-host volatiles for host location by angiosperms-attacking Scolytinae.

It is interesting to note that the compounds found in this study of VOCs from coffee berries and relevant to Scolytinae biology and host finding processes, are not only found in other angiosperm trees e.g., [36], [39], but are also components of the pheromonal systems of some Scolytinae [14] and mammals [34], [35]. Consequently, in evolutionary terms, at the receptor level, the perception of these volatiles by Scolytinae in general appears to be conserved, but behaviorally, individual species may respond differently to different blends of these volatiles. As such, we hypothesise that there must be a common link or a unique origin of all these compounds to enable both temperate- and tropical-occurring bark beetles attacking gymnosperms and angiosperms to incorporate these compounds into their inter- and intraspecific chemical communication systems. We suggest that microorganisms associated with both the host plants and beetles may play a crucial role in the production of these compounds. Indeed, there is undisputable evidence of complex associations between certain Scolytinae and various microorganisms [56]–[62], which may be more widespread than previously known [63].

Recent studies indicate that conophthorin and chalcogran, identified in the present study to mediate host location by the coffee berry borer, are also produced by bacteria [64], and spores of the fungi *Aspergillus* and *Penicillium* spp acting on linoleic and linolenic acid in almonds and pistachio [65]. The authors suggest that conophthorin and chalcogran play an important role in the communication system of the navel orangeworm *Amyelois transitella* (Lepidoptera: Pyralidae), a major pest of almonds and pistachios in California (USA). We cannot exclude the possibility of a similar microbial association with coffee berries, which warrants further research.

With respect to the development of *H. hampei* management strategies, our results have important implications for coffee farmers and researchers. Our finding that the coffee berry borer responds to host and non-host VOCs in its search for a suitable host suggests that a habitat management strategy for coffee production, based on a ‘push-pull’ [66] or a stimulo-deterrent diversionary strategy [67] might be an option to reduce outbreaks an damage by this pest. Thus the ‘pull’ system could be formulated from conophthorin and chalcogran in combination with ethanol and methanol, whereas the ‘push’ system could be derived from α-pinene and verbenone. Instead of the present practice of monocultures, our results support the idea of cultivating coffee intercropped with plants producing conifer monoterpenes compounds that are repellent to *H. hampei* under shade trees similar to the way that coffee naturally grows in the forests of Africa [53], [68].

## Materials and Methods

### General Procedures

Females of the coffee berry borer *H. hampei* were obtained from a stock culture established in July 2005 with beetle-infested coffee berries collected from an organic coffee plantation located in South Kisii (Gucha), Western Kenya (0° 45′ 49.85″ S, 34° 43′ 1.76″ E). The colony was kept at the International Centre of Insect Physiology and Ecology (*icipe*), Nairobi, Kenya on ca. 150 days old coffee berries (*C. arabica* var. Ruiru 11) collected from a plot in a privately owned coffee plantation in Kiambu (Central Province), Kenya (1° 11′ 24.22″ S; 36° 49′ 25.10″E. altitude 1,720 m.a.s.l). No specific permissions were required for the described field study/collections. The field studies did not involve endangered or protected species. The owner of the land gave permission to conduct the study on this site. New *H. hampei* females were regularly collected from the Kiambu plantation and infused into the colony in order to maintain colony vigor. The colony was kept at room temperature (25±1°C), 70% ±5% relative humidity [RH], and a 12∶12 h (L: D) photoperiod. Infested berries were kept inside square plastic containers (40×40×20 cm) with perforated lids (55 mm diameter) covered with insect gauze. The bottom of each container was layered with a 1.5 cm mixture of plaster of Paris and activated charcoal to maintain humidity and to prevent desiccation of the berries and insects [69].

### Collection of Volatile Organic Compounds (VOCs)

Host VOCs were collected from the headspace of organically grown non-infested ca. 150 days old coffee berries (*C. arabica* variety Ruiru 11) obtained from the Kiambu plantation described above. We used coffee berries in the yellow-orange exocarp stage, which is the developmental stage of the berry most attractive to *H. hampei* females in the field [70]. After carefully excising berries directly from coffee tree branches in the field without hand contact, using a sterile scalpel blade No. 21, the berries were placed into a 0.5L sterile cylindrical glass jar with single-port lids (Analytical Research Systems INC, Gainesville, FL, USA), which was covered with aluminum foil and then transported to the laboratory for collection of VOCs. Pre-cleaned (methanol, dichloromethane, pentane, drying) charcoal filters (5 mg; Part No. 91006015; Brechbühler, Schlierensee, Switzerland) were used as absorbent. Each filter was connected by PVC tubing (Masteflex. 06409-15 Tygon mfg by St. Gobain) to a small battery operated pump (PAS-500 Personal Air Sampler, Supelco, Bellefonte, PA, USA), which pulled VOCs-loaded air through the filter at a flow rate of 348 ml/min for 24 hr and a photoregime of 12L:12D. Filters were eluted with 100 µL of GC-grade dichloromethane (Sigma Aldrich, Gillingham, UK), and the eluents were stored at −20°C in 200 µl microtube vial insets placed inside a 1.5 ml glass vial (Sun Sri, TN, USA) with a PTFE lined cap prior to analysis.

In addition, VOCs were collected by SPME, using 3 different commercially available fibers: Polydimethylsiloxane (PDMS), carboxen/PDMS and Carbowax®/divinylbenzene (CW/DVB) which were purchased from Supelco (Supelco Inc. Bellefonte, PA, US).

### Analysis of Volatile Organic Compounds (VOCs)

In order to have *H. hampei* in the right physiological state for electrophysiological analysis, females were collected from berries that had been infested for at least 60 days (J. Jaramillo pers. obs.). Gas chromatography coupled with electroantennographic detection (GC/EAD) was carried out on a Hewlett-Packard (HP) 5890 Series II gas chromatograph equipped with an HP-1 column (30 m×0.32 mm ID x. 0.25 µm, Agilent, Palo Alto, California, USA) using nitrogen as the carrier gas. VOCs were analyzed in the split mode at an injector temperature of 280°C and a split valve delay of 3 min. The oven temperature was held at 35°C for 3 min, programmed at 10°C/min to 280°C and then held at this temperature for 10 min. The column effluent was split 1∶1 for simultaneous recording by flame ionization detector (FID) and EAD. For EAD detection, silver wires in drawn-out glass capillaries filled with Beadle-Ephrussi saline solution [27], and 0.5% polyvinylpyrrolidone served as reference and recording electrodes. The *H. hampei* females were decapitated with a scalpel and the antennae stretched out. Subsequently, the indifferent electrode, from which the tip had been removed, was placed within the head capsule in order to hold it firmly. The first *H. hampei* antennal segment was placed in contact with the recording microelectrode, and a humidified air stream (90–100% RH) passed over the antennal preparation at a flow rate of 1 mL/min. The microelectrodes were connected via an antennal holder to an AC/DC amplifier in DC mode (Syntech, Hilversum, The Netherlands). A GC/EAD program (Syntech GCEAD 2000, Hilversum, The Netherlands) was used to simultaneously record and analyze the amplified EAD and FID signals on a desktop computer. Aliquots (5 µl) of the charcoal-trapped coffee VOCs were analyzed by using female antenna. When testing the complete extract coffee berry VOCs, six antennae per VOCs collection were used and in total 4 individual collections of volatiles from the coffee berries, totaling 24 antennae used for the complet coffee berry VOCs. On the other hand, when synthetic compounds (see below) were tested, 3–12 antennae of females were used per individual compound and dose (Table 2).

Gas chromatography coupled with mass spectrometry (GC/MS) was carried out on an Agilent Technologies 7890A GC linked to a 5795C MS, equipped with MSD ChemStation E.02.00.493, and Wiley 9^th^/NIST 2008 MS Library. Separations were achieved using a HP5 ms column (30 m×0.25 mm iD) under temperature program (5 min 35°C, then 10°C/min to 280°C). Helium served as the carrier gas. We injected 1 µl of the charcoal extract in splitless mode, using helium as the carrier gas at a flow rate of 1 ml/min and based structure assignments of volatiles on the comparison of their mass spectra with those reported in the library mentioned and in the NIST/EPA/NIH Mass Spectral Library 2005a version V2.od, other published spectra [32], [36], and on our own data. SPME-adsorbed VOCs were analysed under the same conditions. Unambiguous structure assignments were based on co-injection with authentic standards.

Enantioselective gas chromatography was carried out by using a 30 m, 0.25 mm fused silica capillary coated with 0.25 mm 2,3-dimethyl-6-*tert*.butyldimethylsilyl-b-cyclodextrin (Macherey & Nagel, Düren, Germany), run under the following conditions: 60°C, then programmed to 150°C at a rate of 10°C/min.

### Chemicals

(5*S*,7*S*)-conophthorin ((5*S*,7*S*)-7-methyl-1,6-dioxaspiro[4.5]decane, purity 99%), *rac*.-conophthorin (purity 97%), 1,6-dioxaspiro[4.5]decane (purity 97%), *rac*.-chalcogran (mixture of the 4 stereoisomers of 2-ethyl-1,6-dioxaspiro[4.4]decane, purity 98%), methyl 3-ethyl-4-methylpentanoate (purity 97%), and 3-ethyl-4-methylpentanol (purity 97%) were synthesized at the University of Hamburg, Germany. Commercial formulations of *rac*.-frontalin (1,5-dimethyl-6,8-dioxabicyclo[3.2.1]octane), verbenone and sulcatol were purchased from ConTech Inc. (USA). All other reference compounds were purchased from Sigma Aldrich Chemical Company (purity ≥ 98%) (Gillingham, Dorset, UK).

### *H. hampei* Responses to Host Volatiles and Selection of Candidate Compounds

In the experiments aiming at elucidating the behavioral responses of *H. hampei* females to VOCs coffee extracts we used two types of devices, e.g. y-tube olfactometer and a windtunnel. In the olfactometer assays we tested a wide range of compounds at different concentrations and based on the results, selected the most attractive compounds to be tested either alone or in mixtures, against blank, against each other or against fresh coffee berries from the field, in the windtunnel assays (see below).

### Y-tube Olfactometer Assays

The behavioral responses of *H. hampei* females were tested to a variety of coffee berry VOCs both *per se* and in some mixtures (Table 2). Criteria for the selection of these compounds were their EAD-activity on the coffee berry borer (conophthorin, 1,6-dioxaspiro[4.5]decane) or a known behavior mediating capacity in other species of Scolytinae (frontalin, chalcogran). We also tested 6-methyl-5-hepten-2-ol (sulcatol), an aggregation pheromone of bark beetles of the genus *Gnathotrichus* spp. [31] because it has been described as a component of the odor bouquet of coffee berries [30]. Methyl 3-ethyl-3-methylpentanoate was included in the tests, because this ester has been described as a semiochemical in ants [40]. Though not eliciting EAD-response, 3-isobutyl-2-methoxy pyrazine and 3-*sec*.-butyl-2-methoxypyrazine were tested, because they were major components of the bouquet of coffee berries, and plant volatiles absolutely crucial in insect plant interactions may sometimes cause very weak EAD-responses from the antennae of the respective insect [71] (W. Francke, pers. observation). Furthermore, following our general idea to develop a pest management system based on push-pull mechanism, we assayed α-pinene and verbenone which are typically known for their repellent activity in other species of Scolytinae [43], [44], [71], [72]. A list of tested compounds is given in table 2.

The aforementioned compounds were tested in a Pyrex glass Y-tube olfactometer (10 mm i.d; stem 85 mm; arms 75 mm at a 60° angle to the stem) (Analytical Research Systems INC, Gainesville, FL, USA). Solutions for each compound were formulated either in dichloromethane or in hexane.

The Y-arms of the olfactometer were attached with PVC tubing (Masteflex. 06409-15 Tygon mfg by St. Gobain) to a sealed glass odor source chamber (internal volume 50 ml) supplied with charcoal-filtered and humidified air (90% RH). The airflow through each arm of the Y-tube was maintained at 260 ml/min by the positive pressure of a battery-powered pump (USDA/ARS-CMAVE, Gainesville, FL, USA). *H. hampei* females were prevented from escaping through the arms of the olfactometer by a screen mesh (1 mm^2^) held with Teflon tape across the openings of each olfactometer arm. Bioassays were carried out in a room (25±1°C; 60% ±5% RH) with diffused uniform fluorescent light (58W). The trials were run between 10∶00 and 17∶00 hrs which according to our experiments coincides with the peak of female activity in the field (J.Jaramillo pers. observation). Females collected from berries infested for 8–12 weeks and starved for 12 hrs before the experiments were individually introduced into the stem of the Y-tube olfactometer and were considered to choose a positive response after spending at least 15 sec beyond the Y-tube intersection into the arm with the tested chemical. Females that failed to choose an arm within 15 min were recorded as non-responders. In total, 15 females (female batch) were used per concentration, and each concentration was tested four times with four different female batches (the total number of females used per concentration and odor source was 60; the total number of females per odor source was 360). The application of odor sources to each arm of the olfactometer was reversed between tests to eliminate directional bias. After each test, the Y-tubes were washed with Teepol® (multipurpose detergent. Teepol® products, Kent, UK), rinsed with acetone followed by distilled water and dried in an oven (100°C) for at least 1 h to remove any volatile contaminants. For each concentration tested, 10 µl of extract were applied to a filter paper strip (30×30 mm), and the solvent was allowed to evaporate for 30 sec before placing it into the chamber of the odor source (see above). In the control arm a paper strip (30×30 mm) loaded with 10 µl of the solvent was used. The concentrations that elicited the highest positive response were used to prepare mixtures for further assays in the wind tunnel.

Although the pyrazines did not elicit any positive response during the GC/EAD analysis, preliminary dose response olfactometer experiments showed some positive reactions of *H. hampei* to 2-methoxy-3-methylpyrazine. Response to 25 ng of this compound was 68.3-26.6% to test vs. control. Therefore, it was decided to include this compound at a concentration of 25 ng in the mixtures used for wind tunnel experiments.

### Wind Tunnel Assays

**Figure 8:**
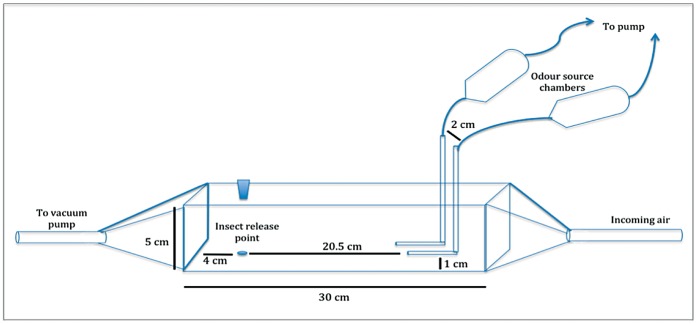
Sketch of the wind tunnel used in our experiments.

To obtain additional information about the behavior of *H. hampei* against selected compounds and mixtures (see above), and to re-check results obtained in the y-tubre olfactometer, bioassays were conducted in a wind tunnel (30×5×5 cm. approx. 750 ml). The tunnel was constructed using transparent Perspex acrylic (Fig. 8) and both ends of the tunnel were fitted with square based pyramids of Perspex acrylic connected to air supply and suction, respectively. A hole (1.2 cm diameter) was drilled at the top of the wind tunnel near one of its ends to introduce the insects. Two L-shaped stainless steel jet pipe-tubing dispensers (Supelco Analytical, Bellefonte, Pennsylvania, USA) (i.d 1.02 mm) were slotted in (2 cm distance between them) from the top of the wind tunnel and were used to dispense the odors. Both L-shaped jet pipes were placed at 1 cm height from the bottom of the wind tunnel (Fig. 8). Each of the jet pipes was attached with PVC tubing (Masteflex. 06409-15 Tygon mfg by St. Gobain) to sealed glass odor source chambers (50 ml) (ARS, Gainesville, FL, USA) supplied with charcoal-filtered and humidified air (90% RH) and through a three airway flowmeter delivery system (Orangeburg, NY, USA). Two of the flowmeters were connected to the odor source holding chambers, and the third one connected to the air inlet port at one of the pyramidal ends of the wind tunnel. Airflow from the chambers into the wind tunnel was maintained at 25.5 ml/min at each dispenser. To ensure a smooth distribution of VOCs and to avoid build up of odors inside the wind tunnel, the airflow in the air inlet was maintained at 100 ml/min by the pressure of a battery-powered pump (USDA/ARS-CMAVE, Gainesville, FL, USA).

Before releasing any compounds, charcoal-cleaned air was passed through the wind tunnel for 10–15 min to remove any potential odor contamination. A wall-mounted exhaust fan was operated before and between the experiments to remove contaminated air from the room. Bioassays were carried out in a dark room (25±1°C; 60% ±5% RH) with a small red fluorescent light (11W), installed 80 cm above the wind tunnel. Similar to the olfactometer assays, the wind tunnel experiments were run only between 10∶00 and 17∶00 hrs to coincide with the peak of *H. hampei* female activity. Females (8–12 weeks old), which had been starved for 12 hrs were used for the bioassays. Females were released on to the mid-line of the tunnel, 20.5 cm apart from the jet pipes, and those that failed to follow the odor source (either flying or walking) or did not display any activation sign within 7 min were recorded as non-responders. For each compound or mixture of compounds tested, 10 µl of the stimulus were applied on a filter paper strip (30×30 mm), allowing the solvent to evaporate for 30 sec before placing the filter paper into the glass odor source chambers. A new stimulus impregnated filter paper was used for each replicate (15 females). The control was a paper strip (30×30 mm) loaded with 10 µl of the solvent. A list of compounds tested during the first round of wind tunnel assays is presented in Table S3.

Subsequently, mixture 4 (see Table 3) was tested against the compounds that had been found to be the most attractive in the y-tube tests: (5*S*,7*S*)-conophthorin alone at both 10 and 35 ng/µl, *rac*.-chalcogran at a concentration 50 ng/µl, and a mixture of (5*S*,7*S*)-conophthorin (35 ng/µl)+*rac*.-chalcogran (50 ng/µl)+ethanol (300 ng/µl)+methanol (700 ng/µl). The commercial dose of an ethanol+methanol mixture used for trapping *H. hampei* in the field is 300 and 700 ng, respectively. Furthermore, the most attractive compounds or blends were selected and tested in the wind tunnel against freshly collected non-infested coffee berries of 150 days of development (the most attractive stage of berries), to check whether the most attractive compounds selected during previous behavioral assays were indeed more attractive than the fresh coffee berries. In total, eight compounds/blends were tested against coffee berries, i.e. (5*S*,7*S*)-conophthorin alone at both 10 and 35 ng/µl, *rac*.-chalcogran at 50 ng/µl, a mixture of (5*S*,7*S*)-conophthorin (35 ng/µl)+*rac*.-chalcogran (50 ng/µl)+ethanol (300 ng/µl)+methanol (700 ng/µl), and mixture 4 ((5*S*,7*S*)-conophthorin 100%+methyl 3-ethyl-4-methylpentanoate 4.3%+*rac*.-chalcogran 3.2% +1,6-dioxaspiro[4.5]decane 2.4%). Moreover, the efficacy of mixture 4 against coffee berries was evaluated without 1,6-dioxaspiro[4.5]decane, without methyl 3-ethyl-4-methylpentanoate, or without 1,6-dioxaspiro[4.5]decane and methyl 3-ethyl-4-methylpentanoate. A total of 225 females were used in the wind tunnel assays (15 individual runs each with a batch of 15 females per compound/mixture).

**Table 3:** Chemicals, blends and concentrations used for behavioral windtunnel assays with *Hypothenemus hampei* females.

Chemicals - concentration	Abbreviation
Ethanol (30****ng/µl)+methanol (70****ng/µl)	Ethanol - Methanol
(5S,7*S*)-Conophthorin (35****ng/µl)+*rac*.-chalcogran (50****ng/µl)	Scon - Chalc
(5S,7*S*)-Conophthorin (35****ng/µl)+*rac*.-chalcogran (50****ng/µl)+ethanol (300****ng/µl)+methanol (700****ng/µl)	35Scon-50Chalc-300Eth-700Meth
(5S,7*S*)-Conophthorin (75****ng/µl)+*rac*.-chalcogran (50****ng/µl)+ethanol (30****ng/µl)+methanol (70****ng/µl)	75Scon-50Chalc-30Eth-70Meth
(5S,7*S*)-Conophthorin (35****ng/µl)+methyl 3-ethyl-4-methylpentanoate (1.5****ng/µl)+*rac*.-chalcogran (2****ng/µl)+1,6-dioxaspiro[4.5]decane (1****ng/µl))	Mixture 4
(5S,7*S*)-Conophthorin (35****ng/µl)+methyl 3-ethyl-4-methylpentanoate (1.5****ng/µl)+*rac*.-chalcogran (2****ng/µl)+1,6-dioxaspiro[4.5]decane (1****ng/µl))+ethanol (300****ng/µl)+methanol (700****ng/µl)	Mixture 4 plus ethanol and methanol

### Statistical Analysis

The entries into treated (compound) or control (solvent) arms of the olfactometer per concentration or per odor source, or reaching the odor source (or control) in the windtunnel trials were compared using χ^2^ test [73]. The number of non-responding *H. hampei* females was not included in the analysis, and so the test was carried out with number of insects per trial (N) minus number of non-responders (NR). The number of responding females used to carry out individual analyses is indicated in the results section. Differences in time *H. hampei* females spent making a choice (odor source or control) in olfactometer and wind tunnel assays were analyzed using the general linear model (GLM) procedure of SAS [73].

## Supporting Information

Figure S1 Plotted 70 eV EI-mass spectrum of 1,6-dioxaspiro[4.5]decane. Some diagnostic fragments are marked by their masses.
(DOC)

Table S1 Results of χ^2^ choice experiments for *Hypothenemus hampei* females responding to different compounds in behavioral olfactometer tests.
(DOCX)

Table S2 Results of χ^2^ choice experiments for *Hypothenemus hampei* females responding to different compounds and blends in behavioral windtunnel tests.
(DOCX)

Table S3 Results of χ^2^ choice experiments for *Hypothenemus hampei* females responding to different compounds/blends against fresh yellow coffee berries from the field.
(DOCX)
