# Supplementary material for: Coffee Berry Borer Joins Bark Beetles in Coffee Klatch
Source: PLoS One. 2013 Sep 20;8(9):e74277. doi: 10.1371/journal.pone.0074277 (PMC3779205; doi:10.1371/journal.pone.0074277)
Supplement: Table S1 — Results of χ2 choice experiments for Hypothenemus hampei females responding to different compounds in behavioral olfactometer tests. (DOCX) [file pone.0074277.s002.docx]

**SI1**.

| Chemicals | χ^2^ Test concentration (ng/μl) | | | | | |
| --- | --- | --- | --- | --- | --- | --- |
|  | 10 | 25 | 50 | 75 | 100 | 200 |
| (7*S*)-conophthorin | χ2 _1_= 18; *P* < 0.001 | χ2 _1_= 10; *P* 0.0016 | χ2 _1_=3.93; *P* 0.0474 | χ2 _1_=11.11; *P* 0.0009 | χ2 _1_= 1.08; *P* 0.2967 | χ2 _1_=8.10; *P* 0.0044 |
| *rac*.-conophthorin | χ2 _1_=0.56; *P* 0.4561 | χ2 _1_= 1.26; *P* 0.2623 | χ2 _1_= 0.026; *P* 0.8728 | χ2 _1_=23.06; *P* < 0.001 | χ2 _1_= 8.76; *P* 0.0031 | χ2 _1_=3.80; *P* 0.0516 |
| Methyl-3-ethyl-4-methylpentanoate | χ2 _1_= 1.07; *P* 0.3017 | χ2 _1_= 11.66; *P* 0.0006 | χ2 _1_= 1.42; *P* 0.2332 | χ2 _1_= 8.97; *P* 0.0028 | χ2 _1_= 0.62; *P* 0.4302 | χ2 _1_= 2.20; *P* 0.1380 |
| 1,6-dioxaspiro[4.5]decane | χ2 _1_= 1.50; *P* 0.2249 | χ2 _1_= 4.41; *P* 0.0356 | χ2 _1_= 0.16; *P* 0.6911 | χ2 _1_= 4.27; *P* 0.03789 | χ2 _1_= 4.90; *P* 0.0269 | χ2 _1_= 4.41; *P* 0.03567 |
| Frontalin | χ2 _1_= 0.58; *P* 0.4458 | χ2 _1_= 0.40; *P* 0.5271 | χ2 _1_= 1.20; *P* 0.2743 | χ2 _1_= 1.53; *P* 0.2170 | χ2 _1_= 0.90; *P* 0.3428 | χ2 _1_= 1.88; *P* 0.17 |
| *rac*.-chalcogran | χ2 _1_=14.30; *P* 0.0002 | χ2 _1_=16.0; *P* < 0.001 | χ2 _1_= 26.95; *P* < 0.001 | χ2 _1_= 12.90; *P* 0.0003 | χ2 _1_= 16.90; *P* < 0.001 | χ2 _1_= 0.133; *P* < 0.7150 |
| 3-Isobutyl-2-methoxypyrazine | χ2 _1_= 1.32; *P* 0.2513 | χ2 _1_= 4.26; *P* 0.039 | χ2 _1_= 0.80; *P* 0.3711 | χ2 _1_= 0.047; *P* 0.8273 | χ2 _1_= 0.43; *P* 0.5127 | χ2 _1_= 0=1.80; *P* 0.1797 |
| 3-*sec*-Butyl-2-methoxypyrazine | χ2 _1_= 7.08; *P* 0.0078 | χ2 _1_= 3.50; *P* 0.0614 | χ2 _1_= 0.22; *P* 0.6394 | χ2 _1_= 7.35; *P* 0.0067 | χ2 _1_= 015; *P* 0.695 | χ2 _1_= 0.07; *P* 0.7855 |
