# Supplementary material for: Coffee Berry Borer Joins Bark Beetles in Coffee Klatch
Source: PLoS One. 2013 Sep 20;8(9):e74277. doi: 10.1371/journal.pone.0074277 (PMC3779205; doi:10.1371/journal.pone.0074277)
Supplement: Table S2 — Results of χ2 choice experiments for Hypothenemus hampei females responding to different compounds and blends in behavioral windtunnel tests. (DOCX) [file pone.0074277.s003.docx]

**SI2.**

| Chemicals | χ^2^ Test | | |
| --- | --- | --- | --- |
|  | df | χ^2^ | *P* |
| Ethanol (30 ng/μl) + methanol (70 ng/μl) | 1 | 11.76 | 0.0006 |
| (7*S*)-Conophthorin (35 ng/μl) + *rac*.-chalcogran (50 ng/μl) | 1 | 4.57 | 0.00325 |
| (7*S*)-Conophthorin (35 ng/μl) + *rac*.-chalcogran (50 ng/μl) + ethanol (300 ng/μl) + methanol (700 ng/μl) | 1 | 23.11 | < 0.0001 |
| (7*S*)-Conophthorin (75 ng/μl) + *rac*.-chalcogran (50 ng/μl) + ethanol (30 ng/μl) + methanol (70 ng/μl) | 1 | 7.143 | 0.0075 |
| Mixture 4: (7*S*)-conophthorin (35 ng/μl) + methyl 3-ethyl-4-methylpentanoate (1.5 ng/μl) + *rac*.-chalcogran (2 ng/μl) + 1,6-dioxaspiro[4.5]decane (1 ng/μl) | 1 | 44.60 | < 0.0001 |
| Mixture 4: (7*S*)-conophthorin (35 ng/μl) + methyl-3-ethyl-4-methylpentanoate (1.5 ng/μl) + *rac*.-chalcogran (2 ng/μl) + 1,6-dioxaspiro[4.5]decane (1 ng/μl)) + ethanol (300 ng/μl) + methanol (700 ng/μl) | 1 | 51.96 | < 0.0001 |
