# Supplementary material for: Coffee Berry Borer Joins Bark Beetles in Coffee Klatch
Source: PLoS One. 2013 Sep 20;8(9):e74277. doi: 10.1371/journal.pone.0074277 (PMC3779205; doi:10.1371/journal.pone.0074277)
Supplement: Table S3 — Results of χ2 choice experiments for Hypothenemus hampei females responding to different compounds/blends against fresh yellow coffee berries from the field. (DOCX) [file pone.0074277.s004.docx]

**SI3.**

| Compound / blend | χ^2^ Test | | |
| --- | --- | --- | --- |
|  | df | χ^2^ | *P* |
| (7*S*)-Conophthorin (35 ng/μl) + *rac*.-chalcogran (50 ng/μl) + ethanol (300 ng/μl) + methanol (700 ng/μl) | 1 | 40.39 | < 0.0001 |
| (7*S*)-Conophthorin (35 ng/μl) | 1 | 32.64 | < 0.0001 |
| ((7*S*)-Conophthorin (10 ng/μl) | 1 | 5.92 | 0.0150 |
| *rac*.-chalcogran (50 ng/μl) | 1 | 13.31 | 0.0003 |
| Mixture 4: (7*S*)-conophthorin (35 ng/μl) + methyl-3-ethyl-4-methylpentanoate (1.5 ng/μl) + *rac*.-chalcogran (2 ng/μl) + 1,6-dioxaspiro[4.5]decane (1 ng/μl) | 1 | 28.97 | < 0.0001 |
| Mixture 4 without 1,6-dioxaspiro[4.5]decane: (7*S*)-conophthorin (35 ng/μl) + methyl-3-ethyl-4-methylpentanoate (1.5 ng/μl) + *rac*.-chalcogran (2 ng/μl) | 1 | 41.98 | < 0.0001 |
| Mixture 4 without methyl 3-ethyl-4-methylpentanoate: (7*S*)-conophthorin (35 ng/μl) + *rac*.-chalcogran (2 ng/μl) + 1,6-dioxaspiro[4.5]decane (1 ng/μl) | 1 | 15.70 | < 0.0001 |
| Mixture 4 without methyl 3-ethyl-4-methylpentanoate and 1,6-dioxaspiro[4.5]decane: ((7*S*)-conophthorin (35 ng/μl) + *rac*.-chalcogran (2 ng/μl)) | 1 | 19.08 | < 0.0001 |
