# Supplementary material for: Coffee Berry Borer Joins Bark Beetles in Coffee Klatch
Source: PLoS One. 2013 Sep 20;8(9):e74277. doi: 10.1371/journal.pone.0074277 (PMC3779205; doi:10.1371/journal.pone.0074277)
Supplement: Supplementary file 4 [file pone.0074277.s001.doc]

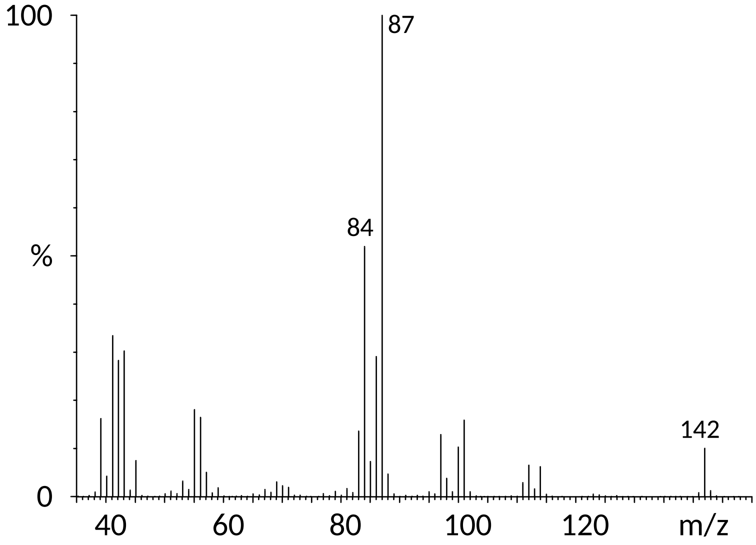
100
%
0
87
84
142
40
60
80
100
120
m/z
